# Accurate Localization of First and Second Heart Sounds via Template Matching in Forcecardiography Signals

**DOI:** 10.3390/s24051525

**Published:** 2024-02-27

**Authors:** Jessica Centracchio, Salvatore Parlato, Daniele Esposito, Emilio Andreozzi

**Affiliations:** Department of Electrical Engineering and Information Technologies, University of Naples Federico II, Via Claudio, 21, I-80125 Naples, Italy; jessica.centracchio@unina.it (J.C.); salvatore.parlato@unina.it (S.P.)

**Keywords:** heart sounds, forcecardiography, template matching, normalized cross-correlation, heart rate variability

## Abstract

Cardiac auscultation is an essential part of physical examination and plays a key role in the early diagnosis of many cardiovascular diseases. The analysis of phonocardiography (PCG) recordings is generally based on the recognition of the main heart sounds, i.e., S1 and S2, which is not a trivial task. This study proposes a method for an accurate recognition and localization of heart sounds in Forcecardiography (FCG) recordings. FCG is a novel technique able to measure subsonic vibrations and sounds via small force sensors placed onto a subject’s thorax, allowing continuous cardio-respiratory monitoring. In this study, a template-matching technique based on normalized cross-correlation was used to automatically recognize heart sounds in FCG signals recorded from six healthy subjects at rest. Distinct templates were manually selected from each FCG recording and used to separately localize S1 and S2 sounds, as well as S1–S2 pairs. A simultaneously recorded electrocardiography (ECG) trace was used for performance evaluation. The results show that the template matching approach proved capable of separately classifying S1 and S2 sounds in more than 96% of all heartbeats. Linear regression, correlation, and Bland–Altman analyses showed that inter-beat intervals were estimated with high accuracy. Indeed, the estimation error was confined within 10 ms, with negligible impact on heart rate estimation. Heart rate variability (HRV) indices were also computed and turned out to be almost comparable with those obtained from ECG. The preliminary yet encouraging results of this study suggest that the template matching approach based on normalized cross-correlation allows very accurate heart sounds localization and inter-beat intervals estimation.

## 1. Introduction

Cardiac auscultation, i.e., listening to heart sounds via a stethoscope, has long been practiced by physicians as an essential part of physical examination [1,2]. Several pathological conditions, such as valvular stenosis or regurgitation, abnormalities in heart rhythm, or heart failure, may be detected via auscultation well before the appearance of any symptoms. For this reason, the assessment of the heart sounds plays a key role in the early diagnosis of many cardiovascular diseases (CVDs) [1,2,3,4].

The origin of the heart sounds has been identified in the motion of the heart valves, the contraction and relaxation of the cardiac muscle, pressure variations in the heart cavities, and the flow of blood through the heart and great vessels during the cardiac cycle. These events cause mechanical vibrations that are transmitted to the chest surface, where they can be heard. Generally, two physiological heart sounds can be auscultated from a healthy adult. They mainly capture the high-frequency and high-amplitude vibrations produced by the heart valves activity. Specifically, the first physiological heart sound, commonly referred to as “S1”, is generated by the closure of the mitral and tricuspid valves and the subsequent opening of the semilunar valves at the onset of ventricular systole, while the second physiological heart sound, commonly referred to as “S2”, is caused by the closure of the aortic and pulmonary valves at the onset of ventricular diastole [1,2,3,4,5,6]. Most of the information content of S1 and S2 is found at frequencies lower than 150 Hz [3]. Occasionally, additional heart sounds, due to low-pitch and low-intensity vibrations of cardiac structures, can be heard. The third heart sound (S3) is attributed to vibrations of the ventricular walls, which are produced by blood deceleration when the ventricles reach their limit of distensibility at the end of rapid filling. S3 is commonly auscultated in young people, but it could be a sign of different CVDs, such as heart failure, mitral stenosis, aortic regurgitation and more, above the age of 40 years. The fourth heart sound (S4) is caused by atrial contraction and blood flowing through the valvular orifice in late ventricular diastole. The presence of S4 is usually associated with a severe pathological condition, such as coronary artery disease, left ventricular hypertension, ischemic heart disease, etc. Apart from S1, S2, S3 and S4, other acoustic phenomena originating from blood flow turbulences, known as murmurs, can be auscultated. Pathological murmurs are very useful in detecting valvular dysfunctions, like stenoses and regurgitations [1,2,3,4,5,6].

Despite its great diagnostic capability, cardiac auscultation is a qualitative examination, which strongly depends on the hearing acuity and the expertise of the physician [1,3,4]. To overcome these limitations, some techniques have been proposed that apply frequency shifts to heart sounds, in order to move their energy to a frequency band corresponding to superior human ear sensitivity [7,8,9]. Phonocardiography (PCG) is the method of recording the heart sounds by means of electronic stethoscopes. This technique retrieves the diagnostic significance of cardiac auscultation while obviating the problem of the subjectivity related to the human hearing sense. PCG enables not only the amplification, digitization, storage, and visualization of the heart sounds, but also the recording of vibrations that cannot be perceived by the human ear. A more effective discrimination between physiological and pathological heart sounds, as well as between heart sounds and murmurs, can be accomplished through the visual inspection of PCG signals, and different types of murmurs can also be distinguished. In addition, information on the timing of the heart sounds with respect to the cardiac cycle, as well as measurements of their intensity, frequency, and duration, can be obtained from the analysis of PCG signals. The correlation between variations of these parameters and different pathological conditions could be used to provide a valuable aid in the diagnosis of many CVDs [2,3,6,10,11,12,13,14,15,16]. A challenging task in PCG signals analysis is the localization of the heart sounds, especially S1 and S2. Because stethoscopes are sensitive to environmental noises and other sounds from the human body (e.g., respiratory sounds, lung sounds, rumbling of the stomach and intestine), denoising is strongly required to improve the accuracy of heart sounds localization [10,17,18,19,20,21]. Several techniques, such as short-time Fourier transform, fast Wavelet transform, tunable-Q Wavelet transform, and S transform, have been used to accomplish this task [17,18,19,20,21,22,23,24,25,26,27]. Different approaches have been proposed in the literature for heart sounds localization. Most of these methods take advantage of simultaneous Electrocardiography (ECG) tracing as a reference signal. However, in the last two decades, research has focused on the development of automated algorithms that perform heart sounds localization without using any reference signal. These tools can be essentially categorized into envelogram-based methods and artificial intelligence-based methods [10,17,18,19,20,21]. Envelogram-based methods generally extract an envelope from the PCG signal by using a Shannon energy operator [24,28,29], a Hilbert transform [30,31,32], or a Teager–Kaiser energy operator [26], among others [33,34]. A fixed or adaptive threshold is then applied to the envelope to locate the peaks, to therefore identify the boundaries of the signal chunks corresponding to heart sounds. However, the sole thresholding operation often fails to selectively detect peaks related to heart sounds. Nevertheless, the great part of the envelogram-based methods is unable to discriminate S1 and S2 [23,26,29,32], or they need to consider additional criteria such as the typical duration of systole and diastole, to distinguish them [22,24,28,31,33,34].

On the other hand, the artificial intelligence-based methods extract many time-domain, frequency-domain, or time-frequency-domain features from each PCG segment, which are then used to train a classification model. Several machine learning or deep learning algorithms, such as k-nearest neighbor, support vector machine, hidden Markov model, decision tree, k-means clustering, logistic regression, and neural networks, have been implemented to discriminate between S1, S2, S3, and S4 heart sounds, and also to distinguish heart sounds from murmurs [25,27,35,36,37,38,39,40,41,42,43] and to recognize abnormal heart sounds [44,45]. Although they achieve high classification performance, artificial intelligence-based methods are far more complex than envelogram-based algorithms in terms of computational burden and require the a priori knowledge of the heart sounds for labelling PCG segments and training a classifier.

Recently, Forcecardiography (FCG) has been introduced as a novel, non-invasive technique for cardio-respiratory monitoring [46,47,48,49]. FCG records the local forces induced on the chest wall by the mechanical activity of the heart and lungs by means of piezoresistive and piezoelectric force sensors, which have also been used to record sphygmic waves [50]. These sensors are equipped with dome-shaped mechanical couplers to ensure an efficient transmission of the force from human tissues to the sensors active area [46,47]. The wide bandwidth of FCG sensors allows them to monitor respiration [48], infrasonic cardiac vibrations [46,49], and heart sounds, all simultaneously from a single contact point on the chest [47]. This capability supports the accurate estimation of inter-breath and inter-beat intervals [46,47,48], as well as cardiac time intervals, such as the pre-ejection period and the left ventricular ejection time [51,52]. The infrasonic cardiac vibrations captured using FCG can be divided in two components: a low-frequency component related to emptying and filling of heart chambers, and a high-frequency component, related to the opening and closure of heart valves, which also exhibits a very high similarity with accelerometric Seismocardiography (SCG) signals [46,47]. The infrasonic FCG components have also been shown to be affected by respiration, which causes both amplitude modulations and morphology variations [48,52,53]. In [47], a morphological comparison was carried out that confirmed the high similarity between PCG signals and the audible component of FCG signals, both in terms of morphology and acoustic impression.

All measurements and analyses related to events of the cardiac cycle require a fundamental task, i.e., the localization of heartbeats. In cardio-mechanical signals, such as PCG, SCG, and FCG, this task is usually performed with the support of a concurrent ECG tracing, which, however, poses a limitation to the standalone application of these cardio-mechanical monitoring techniques. To address this issue, a template matching algorithm has been proposed for ECG-free heartbeat localization in cardio-mechanical signals [54,55]. A template is selected from the analyzed signal to capture the typical heartbeat morphology and the algorithm evaluates the similarity between the template and the whole signal by calculating the normalized cross-correlation (NCC) function. A high similarity between the template and any signal chunk results in a local maximum of the NCC function, which is assumed as heartbeat marker. The template matching algorithm has been tested on SCG and Gyrocardiography signals from 29 healthy subjects and 100 patients with valvular pathologies. The high accuracy achieved in heartbeat localization, as well as the high correlation and negligible errors obtained in inter-beat intervals estimation, demonstrated that the template matching approach is a very simple, effective, and robust solution for continuous heart rate monitoring via a standalone cardio-mechanical approach. Moreover, the feasibility of heart rate variability (HRV) analysis on the inter-beat intervals obtained by using the ECG-free heartbeat localization method based on template matching was investigated [56]. Many time-domain, frequency-domain, and non-linear HRV indices were computed from the time series of these inter-beat intervals and resulted in very close agreement with those provided by the reference ECG signals.

In this study, the template matching algorithm described above was applied to the HS-FCG signals of healthy subjects to investigate its suitability in localizing and discriminating physiological S1 and S2 heart sounds without using any reference tracing. Then, the temporal locations of S1 and S2 were considered for the estimation of inter-beat intervals. Finally, an HRV analysis was carried out on these intervals. Both the inter-beat intervals estimates and the HRV indices obtained using the proposed approach were compared with those provided using simultaneous ECG recordings.

The article is organized as follows: Section 2 summarizes the measurement setup and data collection procedure adopted for FCG signals acquisition in [47] and describes the proposed template matching approach and the methodology for performance evaluation; Section 3 describes the results of performance evaluation; Section 4 discusses the results and highlights the limitations of the study; Section 5 presents the conclusions of the study and suggests possible future developments.

## 2. Materials and Methods

### 2.1. Measurement Setup and Signal Acquisition

FCG signals acquired in a previous study [47] were considered in this work. Indeed, no measurements were carried out in this study, but a retrospective analysis was conducted instead. The FCG signals were acquired on six healthy subjects, during quiet breathing, by means of an FCG sensor placed on their chest. The FCG sensor was placed approximately on the point of maximal impulse [47,57] of each subject via medical adhesive tape and secured by means of a belt fastened around the thorax. The point of maximal impulse was localized by searching for the point where the FCG signal exhibited the highest amplitude around the fifth intercostal space on the midclavicular line.

ECG lead II tracings have also been acquired concurrently, by means of a multiparameter monitor (Welch-Allyn Propaq Encore model). In this study, such ECG signals were considered as a ground truth for the estimation of inter-beat intervals and HRV indices. The simultaneous acquisitions of FCG and ECG signals have been performed via a National Instruments NI-USB4431 DAQ board (National Instruments Corp., 11500 N Mopac Expwy, Austin, TX 78759-3504, USA) with 24-bit precision and a sampling rate of 10 kHz.

### 2.2. Signal Pre-Processing

**Figure 1:**
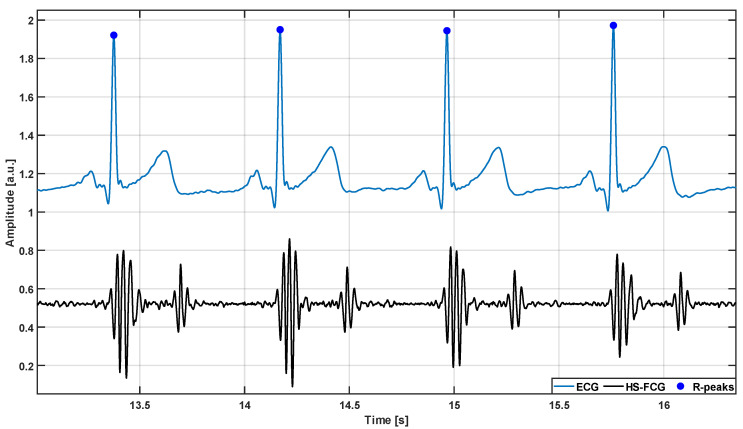
An excerpt of signals from subject #4: ECG (blue line) and heart sounds from FCG (black line). Blue circles mark the locations of R-peaks in the ECG signal.

As in [48,53,54], the respiratory component of the raw FCG sensor signal was first extracted via a Savitzky-Golay filter [58] with frame length approximately corresponding to a time interval of 1.5 s. The respiratory component was then subtracted from the raw FCG sensor signal to obtain the actual FCG signal (cardiac component). The heart sounds component was extracted via a fourth order zero-lag Butterworth band-pass filter with cut-off frequencies of 30–200 Hz, as shown in Figure 1. On the other hand, the ECG signal was first band-pass filtered in the 0.5–40 Hz frequency band via a fourth order zero-lag Butterworth filter. Then, notch filters were used to remove powerline interference and its higher harmonics. Finally, the well-known Pan and Tompkins algorithm [59] implemented in the “*BioSigKit*” MATLAB^®^ toolbox [60] was used to locate the R-peaks (see Figure 1). MATLAB^®^ R2018b (MathWorks, Inc., Natick, MA, USA) was used to perform all processing operations described in this study.

### 2.3. Template Matching Approach

A template-matching technique, previously applied on SCG [54] and Gyrocardiography [55] signals, was applied in this study to separately localize S1 and S2 heart sounds in FCG recordings. The template matching approach consists of three steps, namely template selection, computation of the NCC function between the whole signal and the template previously selected, and the localization of the NCC local maxima. Such local maxima correspond to the temporal locations at which the signal exhibits the local highest similarity with the selected template and are used as timings of the events to be localized. In this study, the template matching approach was used for two purposes, i.e., separately locating S1 and S2 heart sounds with two distinct templates and locating heartbeats by using a single heartbeat template consisting of both heart sounds (S1–S2 pair). Further details on the selection of such templates are provided in the following paragraph.

#### 2.3.1. Templates Selection Criteria

First, templates were identified in each heart sounds recording to capture the typical heart sounds waveforms, which were regarded as a morphological reference. Indeed, two different templates were chosen to separately locate S1 and S2 sounds. In addition, a third template was selected, which included the S1–S2 pair belonging to a single heartbeat.

**Figure 2:**
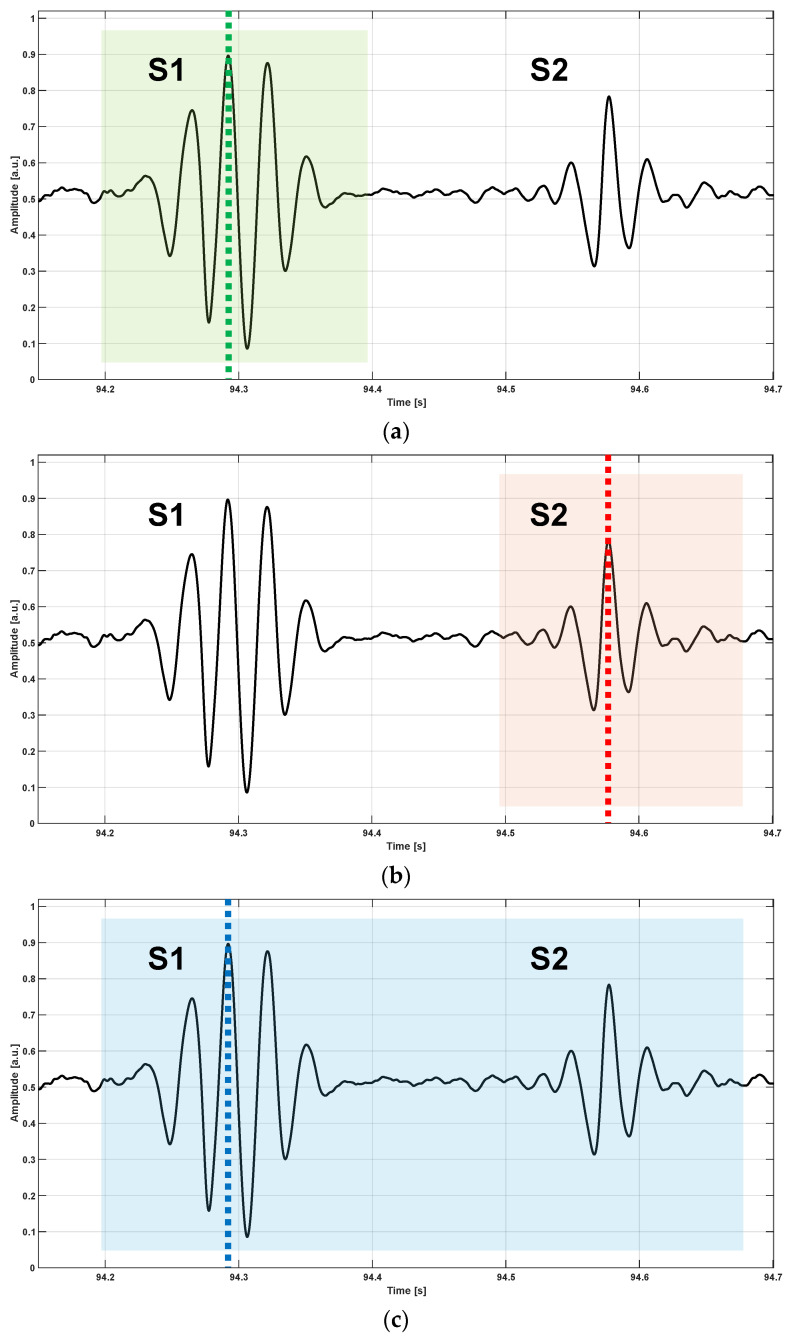
Examples of template selection. (**a**) The portion of the HS-FCG signal selected as a template of S1 sound is enclosed within the light green box, while the green dashed line marks the position of the absolute maximum within the S1 template; (**b**) the portion of the HS-FCG signal selected as a template of S2 sound is enclosed within the light red box, while the red dashed line marks the position of the absolute maximum within the S2 template; (**c**) the portion of the HS-FCG signal selected as a template of S1–S2 pair is enclosed within the light blue box, while the blue dashed line marks the position of the absolute maximum within the S1–S2 template. The positions of the absolute maxima within the templates were used to re-align the corresponding NCC functions.

**Figure 3:**
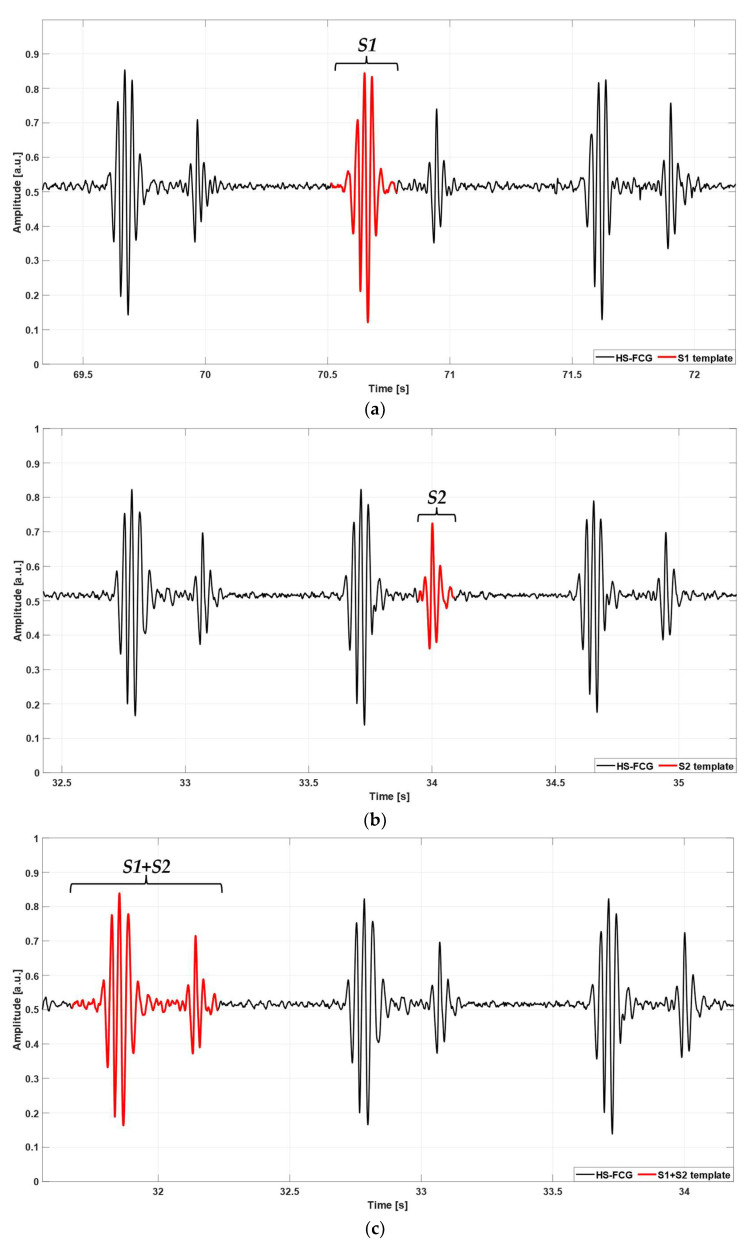
Examples of heart sounds templates (red line) selected in FCG signals of subject #4. (**a**) S1 template, (**b**) S2 template, (**c**) S1–S2 template.

The templates were selected manually, according to the following criteria. The S1 and S2 templates enclosed all the oscillations of the sound and short tracts at the beginning and at the end of the sound. The S1–S2 template enclosed the S1 and S2 sounds, the interval in between, and short tracts at the beginning and at the end of the S1–S2 pair. Examples of selected templates are shown in Figure 2 and Figure 3.

#### 2.3.2. Localization of Single Heart Sounds and Heartbeat Detection

**Figure 4:**
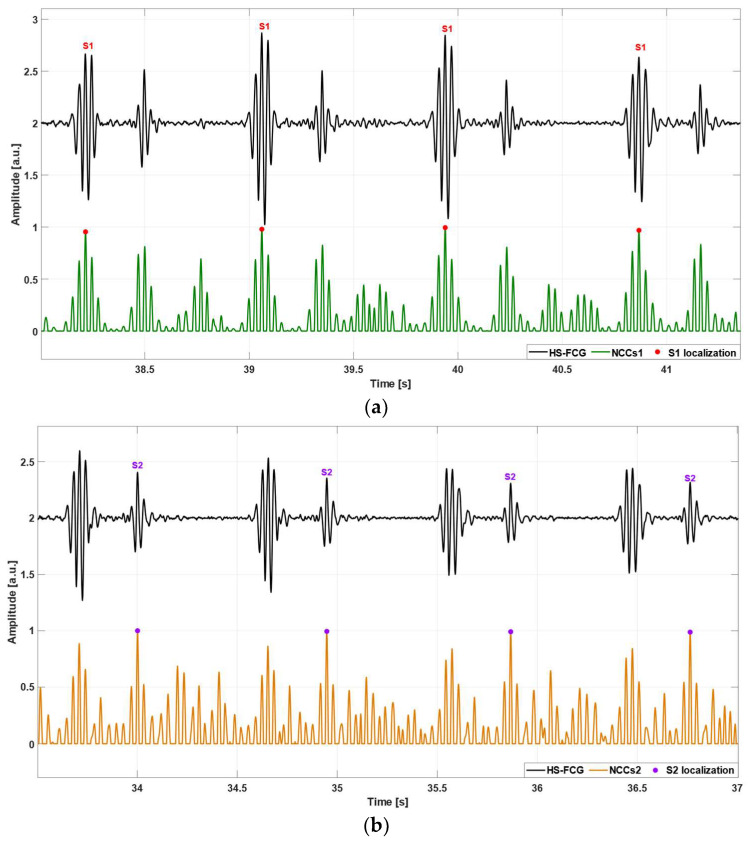

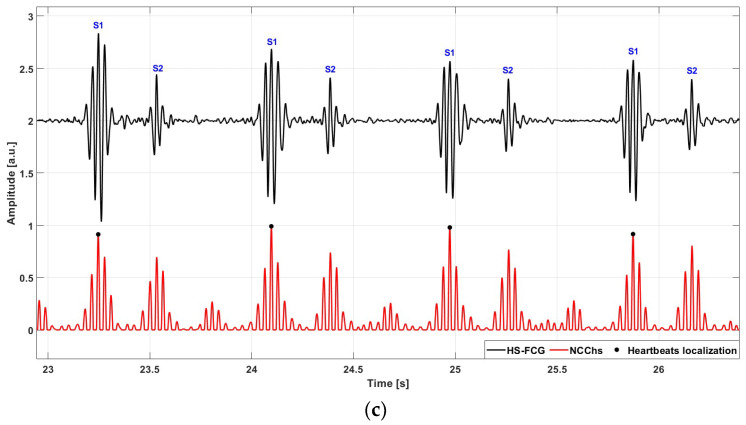
Examples of events localization via the template matching method in FCG signals of subject #4: (**a**) S1 sounds localization: heart sounds signal (black line), NCC function computed by using the S1 template (green line), NCC peaks indicating temporal locations of S1 sounds (red points); (**b**) S2 sounds localization: heart sounds signal (black line), NCC function computed by using the S2 template (orange line), NCC peaks indicating temporal locations of S2 sounds (violet points); (**c**) S1–S2 pairs localization: heart sounds signal (black line), NCC function computed by using the S1–S2 template (red line), NCC peaks indicating temporal locations of S1–S2 pairs (black points). Please note that each NCC function was re-aligned according to the relative position of the absolute maximum within the selected template. In the case of the S1–S2 template selected from the HS-FCG signal of subject #4, the absolute maximum of the S1–S2 template coincided with the S1 peak, therefore, also the NCC peaks in panel (**c**) turned out to be aligned with the S1 peaks in the HS-FCG signal.

The NCC function, as defined in [61,62,63], is considered as a similarity measure between the selected template and signal chunks. Local maxima of the NCC function correspond to signal chunks with the highest local similarity to the template and allow localizing the patterns of interest. The NCC local maxima were detected via the Matlab^®^ function findpeaks. The S1 and S2 templates were used separately to locate the S1 and S2 heart sounds, while the S1–S2 template was used to locate the whole heartbeats (i.e., S1–S2 pairs). Examples of the results obtained by using the S1, S2, and S1–S2 templates are shown, respectively, in Figure 4a–c. Three series of time markers were obtained, i.e., the timings of S1, S2, and S1–S2 pairs.

### 2.4. Performance Analysis

The performances achieved by using the S1 and S2 templates were evaluated via quantitative analyses of their ability to correctly recognize the related events of interest, and to accurately provide the timings of such events. The performances obtained by using the S1–S2 template were also evaluated, since in previous studies on Seismocardiography and Gyrocardiography signals it had been observed that better results could be obtained in heartbeat localization by using templates comprising both systolic and diastolic vibrations.

For the analysis of recognition accuracy, true events (true positives, TPs), missed events (false negatives, FNs), and false events (false positives, FPs) were annotated for each subject and for each template, as in [54,55]. Then, sensitivity and positive predictive value (PPV) were eventually computed, according to the following equations, to provide statistical evaluations of template matching performance:

(1) $$\mathrm{Sensitivity}=\frac{\mathrm{TP}}{\mathrm{TP}+\mathrm{FN}}\cdot 100$$

(2) $$\mathrm{PPV}=\frac{\mathrm{TP}}{\mathrm{TP}+\mathrm{FP}}\cdot 100$$

**Figure 5:**
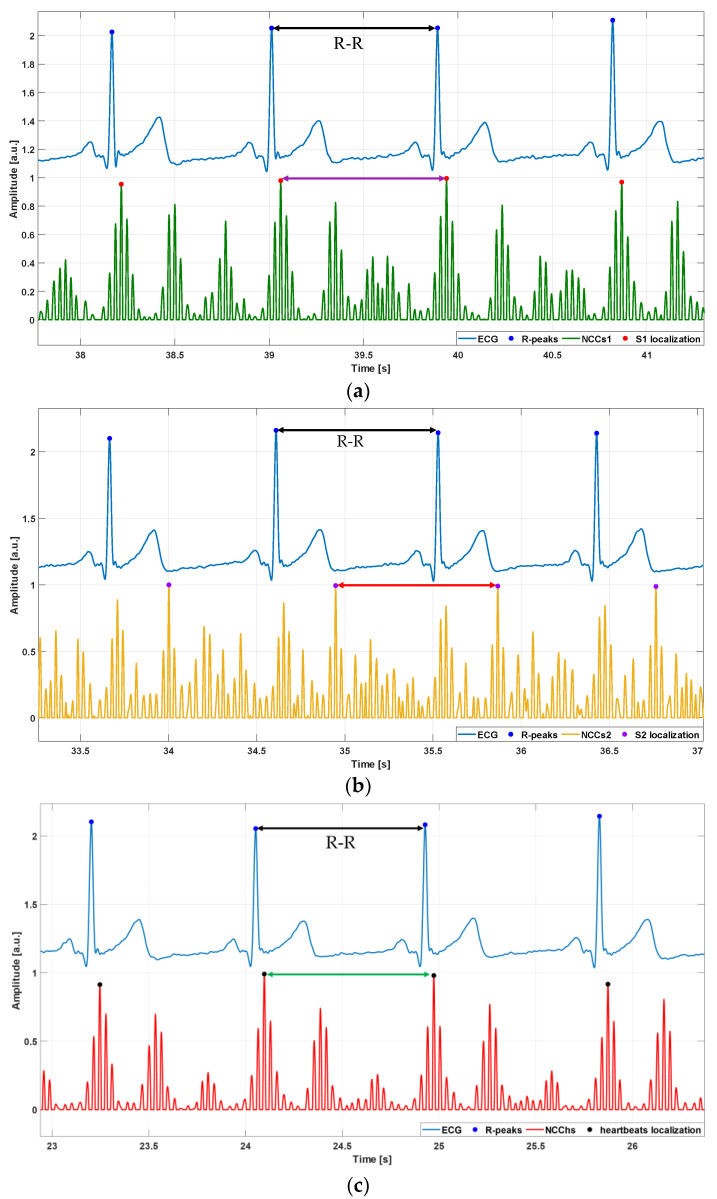
Examples of inter-beat intervals estimation in signals of subject #4. Inter-beat intervals were estimated in the ECG signal as the temporal difference between two consecutive R-peaks (double black arrow), and in the heart sounds signals as the temporal difference between two consecutive peaks in the NCC function obtained via: (**a**) S1 template (double purple arrow); (**b**) S2 template (double red arrow); (**c**) S1–S2 template (double green arrow).

For the analysis of localization accuracy, inter-beat intervals were estimated separately from the series of S1 timings, S2 timings, and S1–S2 timings, as they all mark the presence of heartbeats. The higher the accuracy in estimation of inter-beat intervals, the higher the accuracy in the localization of S1, S2, and S1–S2 events. Inter-beat intervals were computed as time differences between couples of subsequent recognized events. Figure 5 shows some examples of inter-beat intervals estimation from the timings of S1, S2, and S1–S2 pairs. These results were compared with those obtained using ECG (considered as reference) via Passing–Bablok linear regression [64], correlation, and Bland–Altman analyses [65,66]. To this end, the Matlab^®^ functions “Passing-and-Bablok-regression” [67] and “bland-altman-and-correlation-plot” [68] were used. The inter-beat intervals related to missed and false events were removed from the analyses.

### 2.5. Heart Rate Variability Analysis

The heartbeats localization accuracy was further assessed by comparing several HRV indices estimated from the inter-beat intervals series (tachograms) computed from the timings of the S1–S2 pairs and those estimated from ECG. The inter-beat intervals related to missed and false events were included in this analysis. To this aim, the time-domain, frequency-domain, and non-linear HRV indices listed in Table 1 were computed, for each subject, via the well-known software “Kubios HRV Standard” [69,70,71,72]. As in [56], the tachograms were analyzed to detect and correct possible artifacts by following the Kubios HRV guidelines [73]. Linear regression, correlation, and Bland–Altman analyses were carried out to compare the HRV indices obtained from the S1–S2 pairs with those obtained from ECG.

## 3. Results

### 3.1. Recognition and Localization Accuracy

Table 2, Table 3 and Table 4 summarize the results obtained in the recognition of S1 and S2 sounds, and S1–S2 pairs, respectively. In particular, each table outlines the number of events recognized via the template matching method, as well as the number of TPs, FPs, and FNs, and the number of inter-beat intervals considered. The number of inter-beat intervals included depended on the distribution of the missed detections (FN), particularly if there were isolated FNs or multiple subsequent FNs. A total of 1429 cardiac cycles were analyzed. The template matching approach achieved a sensitivity and PPV of 97.9% and 97.6% for S1 recognition, 96.6% and 95.2% for S2 recognition, 87.0% and 91.2% for S1–S2 pairs.

**Figure 6:**
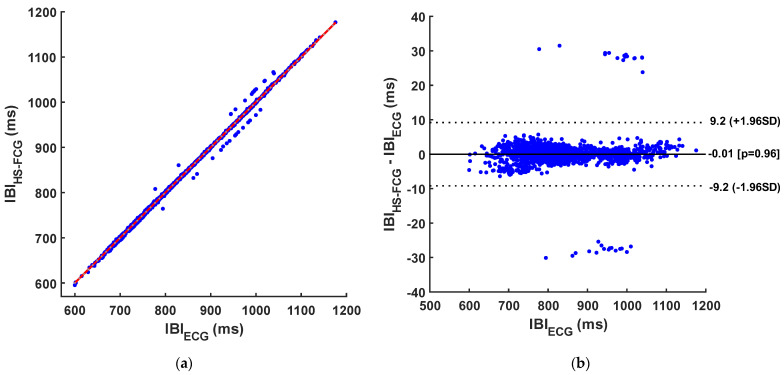
Results of statistical analyses on inter-beat intervals obtained from S1 sounds and from ECG: (**a**) Regression and correlation plot (regression line is depicted in red); (**b**) Bland–Altman plot.

**Figure 7:**
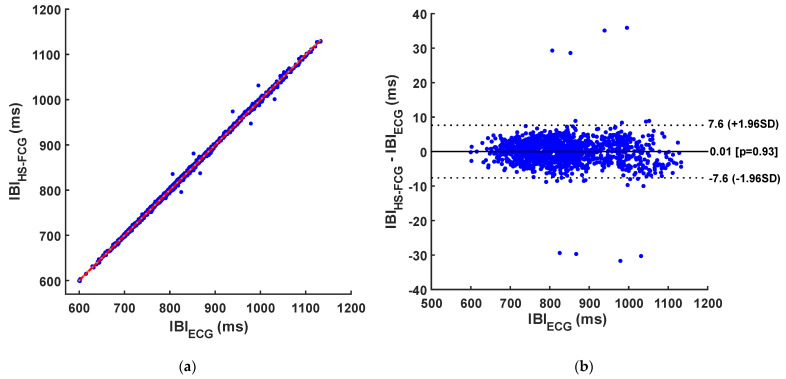
Results of statistical analyses on inter-beat intervals obtained from S2 sounds and from ECG: (**a**) Regression and correlation plot (regression line is depicted in red); (**b**) Bland–Altman plot.

**Figure 8:**
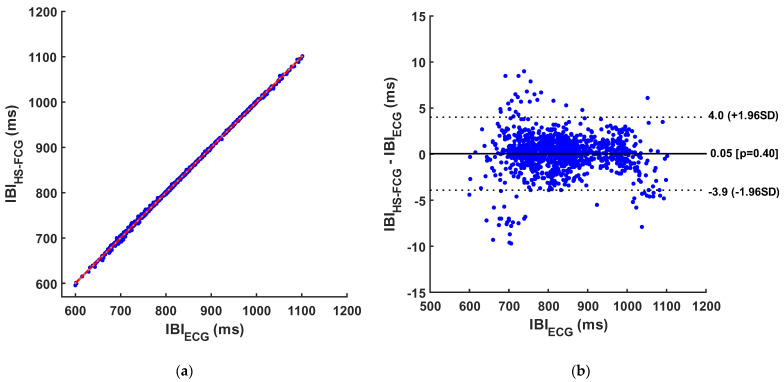
Results of statistical analyses on inter-beat intervals obtained from S1–S2 pairs and from ECG: (**a**) Regression and correlation plot (regression line is depicted in red); (**b**) Bland–Altman plot.

The results of regression, correlation, and Bland–Altman analyses of inter-beat intervals are reported in Table 5 and Table 6 and depicted in Figure 6, Figure 7 and Figure 8. The regression and correlation analyses reported almost unitary slopes and intercepts of about 1 ms, with Pearsons’s r in excess of 0.999. The Bland–Altman analyses reported negligible biases (zero was comprised in the 95% confidence interval of the bias) for all templates, as well as limits of agreement (LoA) of ±9.2 ms for S1, ±7.6 ms for S2, and ±4 ms for S1–S2 pairs.

### 3.2. HRV Indices Extraction

Table 7 and Table 8 outline the results of linear regression, correlation, and Bland–Altman analyses performed on HRV indices extracted from FCG and ECG signals. In particular, the results of linear regression and correlation analyses reported no statistically significant differences from unity for the slopes, no statistically significant differences from zero for the intercepts, and Pearson’s correlation coefficients in excess of 0.8 for all HRV indices. The Passing–Bablok cusum test also confirmed the linearity between all HRV indices extracted from FCG and ECG signals (*p* > 0.1). The results of Bland–Altman analyses reported no statically significant bias on any of the HRV indexes (all the confidence intervals include zero). The HRV indices extracted from each subject are reported in Appendix A (Table A1).

## 4. Discussion

This study proposes the use of the template-matching technique for accurate recognition of heart sounds. In particular, the effectiveness of this technique in separately recognizing S1 and S2 heart sounds was investigated. The template-matching technique has several advantages with respect to other approaches. The approaches based on energy or envelope operators can easily recognize the presence of heart sounds but are usually unable to distinguish between S1 and S2 sounds and are very sensitive to various sources of noise. Instead, the template matching based on normalized cross-correlation is able to recognize the waveform of a specific heart sound because it evaluates the morphological similarity, which also prevents other noises to be incorrectly recognized as heart sounds, regardless of their amplitude. On the other hand, machine learning techniques have a much higher computational load, as opposed to the template-matching technique, which could easily be implemented in real time on microcontrollers, paving the way for long-term patient monitoring applications. It is worth underlining that the proposed technique does not require a synchronously acquired ECG signal: it was only used here for performance evaluation.

The results of this preliminary study are exceptionally encouraging. In fact, the proposed approach proved capable of separately classifying S1 and S2 sounds in more than 96% of all heartbeats. Linear regression, correlation, and Bland–Altman analyses showed that the template matching method allowed the estimation of inter-beat intervals with high accuracy. Indeed, 95% of the estimation errors were confined within 10 ms, which corresponds to relative errors lower than 2% by considering heart rates between 50 and 120 bpm. Further statistical analyses showed that HRV indices were estimated with reasonable accuracy, by achieving mean absolute percentage errors within 8% for all time-domain and non-linear indices, apart from NN50 and pNN50. Higher errors, within 32%, were found for frequency-domain indices.

It is interesting to note that the best results were obtained by considering only the first sound S1. This can be explained by recalling that the second sound undergoes substantial morphological changes in relation to respiration (the well-known physiologic splitting of S2).

### Limitations of the Study

The heart sounds recording considered in this study had been acquired in a quiet environment. The proposed algorithm needs to be further verified in the presence of relevant external noises in order to be adopted as a sound recognizer in applications of long monitoring of patients during the performance of their daily activities.

All the tests were carried out on a limited number of subjects sitting at rest. Studies on a larger cohort of subjects are undoubtedly needed for a proper assessment of this technique, also considering the circumstance that the subject may change posture and may freely move. In such cases, morphological variations of heart sounds might occur, which might make the template-matching technique less effective. However, a dynamic update of the template might be considered to overcome such possible drawbacks.

The cardiac sound recordings considered in this study had been acquired only from healthy subjects: it was not possible to verify the recognition of S1 and S2 sounds in conjunction with other heart sounds (i.e., S3 and S4) or murmurs, which are generally associated with pathologies. Therefore, further tests on pathological subjects are foreseen to thoroughly assess the accuracy of heart sounds recognition via template matching.

## 5. Conclusions

The template matching approach proposed for accurate localization of first and second heart sounds in FCG signals has shown great promise and it could be effective on heart sounds recorded via common electronic stethoscopes. Future studies could evaluate its performance on public databases of heart sounds. However, various electronic stethoscopes are known to suffer from a noticeable sensitivity to external noises, while FCG sensors are, in principle, less sensitive to external noises, being essentially contact microphones without a chest piece bell.

FCG sensors are small, lightweight, nonobstructive, and wearable, therefore they are particularly suitable for a long-term monitoring, which could lead to much more accurate and early diagnosis of various heart diseases [74,75,76,77]. Furthermore, specific cardiac diseases manifest with pronounced S3 and S4 sounds and murmurs. Indeed, murmurs have a higher frequency content than S1 and S2 sounds, while S3 and S4 are low-pitched, low-intensity sounds, which may be difficult to perceive via auscultation, so that chest palpation, i.e., perception of inaudible vibrations, is also recommended. The very large bandwidth of FCG sensors make them very sensitive to both low (subsonic) and high frequency vibrations and sounds, and could greatly help in the detection of S3 and S4 sounds and murmurs. This could be the subject of future studies.

## Figures and Tables

**Table 1:** Time-domain, frequency-domain, and non-linear HRV indices considered in this study.

*Time-Domain Indices*	*Frequency-Domain Indices*	*Non-Linear Indices*
Mean RR (ms)	LF absolute power (ms^2^)	Poincaré SD1
SDNN (ms)	HF absolute power (ms^2^)	Poincaré SD2
Mean HR (bpm)	LF relative power	Poincaré SD2/SD1
SD HR (bpm)	HF relative power	Approximate entropy
Min HR (bpm)	LF normalized power	Sample entropy
Max HR (bpm)	HF normalized power	DFA α1
RMSSD (ms)	Total power (ms^2^)	DFA α2
NN50 (adim)	LF/HF	
pNN50 (adim)		

**Table 2:** Result of S1 sounds detection for each subject. The numbers of TPs, FNs, and FPs are reported. The numbers of inter-beat intervals considered in subsequent analyses are also reported.

Subject ID	TP	FP	FN	Inter-Beat Intervals Analyzed
1	378	0	0	377
2	188	2	3	183
3	158	13	8	150
4	185	20	19	172
5	336	0	0	335
6	154	0	0	153
Total	1399	35	30	1370

**Table 3:** Result of S2 sounds detection for each subject. The numbers of TPs, FNs, and FPs are reported. The numbers of inter-beat intervals considered in subsequent analyses are also reported.

Subject ID #	TP	FP	FN	Inter-Beat Intervals Analyzed
1	378	0	0	377
2	160	44	31	130
3	160	10	6	154
4	196	13	8	190
5	336	0	0	335
6	151	3	3	146
Total	1381	70	48	1332

**Table 4:** Result of S1–S2 pairs detection for each subject. The numbers of TPs, FNs, and FPs are reported. The numbers of inter-beat intervals considered in subsequent analyses are also reported.

Subject ID #	TP	FP	FN	Inter-Beat Intervals Analyzed
1	377	1	1	374
2	163	28	28	133
3	95	37	71	76
4	152	52	52	130
5	303	2	33	272
6	154	0	0	153
Total	1244	120	185	1138

**Table 5:** Results of linear regression and correlation analyses on inter-beat intervals obtained from heart sounds with S1, S2, and S1–S2 pairs, as compared to those obtained from ECG.

*Template*	*Regression and Correlation Analysis*					
	*r*	*CI _r_*	*Slope*	*CI _slope_*	*Intercept (ms)*	*CI _intercept_ (ms)*
S1	0.9991	[0.9990; 0.9992]	1.000	[0.999; 1.002]	0.100	[−1.209; 0.693]
S2	0.9993	[0.9992; 0.9994]	0.998	[0.997; 1.000]	1.442	[0.100; 2.929]
S1–S2	0.9998	[0.9998; 0.9998]	0.999	[0.998; 1.000]	0.851	[0.100; 1.853]

**Table 6:** Results of Bland–Altman analyses on inter-beat intervals obtained from heart sounds with S1, S2, and S1–S2 pairs, as compared to those obtained from ECG.

*Template*	*Bland–Altman Analysis*				
	*bias (ms)*	*CI bias (ms)*	*LoA (ms)*	*CI LoA_min_ (ms)*	*CI LoA_max_ (ms)*
S1	−0.00666	[−0.242; 0.256]	[−9.191; 9.205]	[−9.62; −8.766]	[8.779; 9.630]
S2	0.00970	[−0.219; 0.200]	[−7.633; 7.614]	[−7.991; −7.276]	[7.256; 7.972]
S1–S2	0.0509	[−0.169; 0.0668]	[−4.017; 3.916]	[−4.219; −3.816]	[3.714; 4.117]

**Table 7:** Results of regression and correlation analyses performed on HRV indices obtained from heart sounds and ECG.

*HRV Index*	*r*	*CI _r_*	*Slope*	*CI _slope_*	*Intercept*	*CI _intercept_*
Mean RR *	0.999	[0.994; 0.999]	0.966	[0.928; 1.024]	29.2 ms	[−20.7; 61.8] ms
SDNN *	0.869	[0.194; 0.986]	0.971	[−0.004; 3.476]	0.9 ms	[−96.4; 38.3] ms
Mean HR *	0.999	[0.996; 1.000]	0.964	[0.922; 1.021]	2.54 bpm	[−1.51; 5.47] bpm
SD HR *	0.962	[0.684; 0.996]	0.975	[0.477; 1.457]	0.07 bpm	[−1.68; 1.70] bpm
Min HR *	0.992	[0.921; 0.999]	0.993	[0.791; 1.165]	0.45 bpm	[−11.03; 13.31] bpm
Max HR *	0.999	[0.999; 1.000]	1.015	[0.979; 1.050]	−1.30 bpm	[−4.02; 1.48] bpm
RMSSD *	0.806	[−0.0168; 0.978]	1.111	[0.567; 6.135]	−4.4 ms	[−170.5; 14.5] ms
NN50 *	0.961	[0.678; 0.996]	1.035	[0.400; 4.556]	−7.069	[−87.667; 18.800]
pNN50 *	0.886	[0.264; 0.987]	1.071	[0.211; 6.429]	−0.188	[−76.119; 16.502]
LF absolute power *	0.858	[0.152; 0.984]	0.836	[0.105; 1.687]	127.50 ms^2^	[−412.46; 596.06] ms^2^
HF absolute power *	0.829	[0.0519; 0.981]	0.881	[0.119; 3.897]	29.49 ms^2^	[−1142.50; 497.97] ms^2^
LF relative power *	0.959	[0.664; 0.996]	0.942	[0.124; 1.914]	3.56	[−54.32; 44.30]
HF relative power *	0.957	[0.653; 0.996]	0.943	[−0.0033; 1.708]	2.14	[−24.53; 46.37]
LF normalized power *	0.957	[0.653; 0.996]	0.942	[0.0670; 1.745]	3.58	[−47.10; 48.64]
HF normalized power *	0.958	[0.658; 0.996]	0.943	[0.0632; 1.738]	2.17	[−27.00; 44.63]
Total power *	0.829	[0.0535; 0.981]	0.734	[−0.188; 2.065]	325.24 ms^2^	[−1444.80; 1375.40] ms^2^
LF/HF *	0.997	[0.975; 0.9997]	0.758	[0.0501; 1.847]	0.23	[−1.41; 1.04]
Poincaré SD1 *	0.805	[−0.0186; 0.978]	1.109	[0.565; 6.125]	−3.1 ms	[−120.6; 10.3] ms
Poincaré SD2 *	0.921	[0.434; 0.992]	0.849	[0.268; 1.952]	7.1 ms	[−44.2; 33.2] ms
SD2/SD1 *	0.972	[0.760; 0.997]	0.987	[0.692; 2.500]	0.03	[−2.44; 0.49]
Approximate entropy *	0.968	[0.729; 0.997]	1.031	[0.285; 1.734]	−0.062	[−0.713; 0.598]
Sample entropy *	0.830	[0.0565; 0.981]	0.739	[−0.639; 1.381]	0.409	[−0.678; 3.067]
DFA α1 *	0.900	[0.328; 0.989]	0.966	[0.288; 4.540]	0.037	[−3.608; 0.697]
DFA α2 *	0.996	[0.961; 0.9996]	0.997	[0.836; 1.509]	−0.004	[−0.121; 0.029]

* cusum linearity test *p*-value > 0.1.

**Table 8:** Results of Bland–Altman analyses performed on HRV indices obtained from heart sounds and ECG. In cases of measurement differences with non-normal distribution, the bias was estimated as the median of differences, and the limits of agreement as the 2.5th and 97.5th percentiles.

*HRV Index*	*bias*	*CI _bias_*	*LoA*	*CI _LoA min_*	*CI _LoA max_*
Mean RR * (ms)	0.2	[−5.0; 4.0]	[−10.0; 4.9]	[−10.03; 0.02]	[3.14; 4.90]
SDNN (ms)	−0.2	[−5.6; 5.1]	[−14.5; 14.0]	[−26.51; −2.49]	[2.01; 26.02]
Mean HR (bpm)	−0.03	[−0.42; 0.35]	[−1.05; 0.98]	[−1.90; −0.19]	[0.13; 1.83]
SD HR (bpm)	< 0.01	[−0.31; 0.31]	[−0.82; 0.83]	[−1.51; −0.12]	[0.13; 1.52]
Min HR (bpm)	< 0.01	[−1.05; 1.04]	[−2.76; 2.75]	[−5.08; −0.44]	[0.43; 5.07]
Max HR (bpm)	−0.1	[−0.36; 0.15]	[−0.79; 0.58]	[−1.36; −0.21]	[0.003; 1.155]
RMSSD * (ms)	−0.2	[−2.2; 6.0]	[−2.5; 12.0]	[−2.50; −1.89]	[0.08; 11.98]
NN50	−2.500	[−14.877; 9.877]	[−35.247; 30.247]	[−62.825; −7.669]	[2.669; 57.825]
pNN50	0.227	[−5.590; 6.044]	[−10.637; 11.091]	[−21.212; −0.062]	[0.517; 21.666]
LF absolute power * (ms^2^)	9.19	[−616.34; 91.13]	[−1041.70; 151.53]	[−1041.70; −190.96]	[30.73; 151.53]
HF absolute power * (ms^2^)	−21.06	[−83.34; 150.77]	[−121.71; 296.36]	[−121.71; −44.97]	[5.18; 296.36]
LF relative power	−1.24	[−6.99; 4.51]	[−16.45; 13.97]	[−29.25; −3.64]	[1.16; 26.78]
HF relative power	1.11	[−4.87; 7.09]	[−14.71; 16.93]	[−28.03; −1.39]	[3.61; 30.25]
LF normalized power	−1.25	[−7.31; 4.82]	[−17.30; 14.80]	[−30.81; −3.78]	[1.29; 28.32]
HF normalized power	1.21	[−4.79; 7.20]	[−14.65; 17.07]	[−28.01; −1.30]	[3.71; 30.42]
Total power (ms^2^)	−157.90	[−702.70; 386.90]	[−1599.30; 1283.50]	[−2813.20; −385.40]	[69.59; 2497.40]
LF/HF *	−0.05	[−2.41; 0.13]	[−4.18; 0.23]	[−4.18; −0.63]	[0.03; 0.23]
Poincaré SD1 * (ms)	−0.2	[−1.5; 4.3]	[−1.8; 8.5]	[−1.75; −1.34]	[0.06; 8.49]
Poincaré SD2 * (ms)	0.1	[−6.1; 3.7]	[−12.0; 7.0]	[−12.00; −0.14]	[0.30; 7.01]
SD2/SD1	−0.06	[−0.22; 0.10]	[−0.49; 0.37]	[−0.85; −0.13]	[0.01; 0.74]
Approximate entropy	−0.017	[−0.068; 0.034]	[−0.153; 0.119]	[−0.267; −0.038]	[0.004; 0.233]
Sample entropy	−0.004	[−0.223; 0.215]	[−0.583; 0.575]	[−1.071; −0.096]	[0.087; 1.062]
DFA α1 *	0.004	[−0.177; 0.015]	[−0.237; 0.020]	[−0.237; −0.116]	[0.011; 0.020]
DFA α2	−0.007	[−0.016; 0.002]	[−0.031; 0.017]	[−0.051; −0.011]	[−0.004; 0.037]

* Non-normal distribution of differences.

## Data Availability

The datasets presented in this article are not readily available because informed consent from the subjects involved was obtained only for this study and not for public availability. Requests to access the datasets should be directed to E.A. (emilio.andreozzi@unina.it).

## Appendix A

**Table A1:** HRV indices estimated for each subject.

*HRV Indices*	*Unit*	*Sub #1*		*Sub #2*		*Sub #3*		*Sub #4*		*Sub #5*		*Sub #6*	
		*ECG*	*HS*	*ECG*	*HS*	*ECG*	*HS*	*ECG*	*HS*	*ECG*	*HS*	*ECG*	*HS*
Mean RR	ms	799.929	800.061	971.447	974.584	1022.094	1012.067	868.063	868.413	733.709	738.605	844.493	844.512
SDNN	ms	38.991	39.091	29.420	37.001	46.792	38.163	49.056	49.136	38.733	38.194	21.746	21.693
Mean HR	bpm	75.006	74.994	61.764	61.565	58.703	59.285	69.119	69.092	81.776	81.234	71.049	71.047
SD HR	bpm	3.685	3.690	1.902	2.382	2.691	2.243	3.945	4.006	4.365	4.298	1.841	1.837
Min HR	bpm	66.844	66.860	57.332	55.777	52.804	54.387	60.505	60.478	73.668	73.627	67.230	67.231
Max HR	bpm	88.283	88.279	68.382	68.398	64.295	63.754	80.483	80.248	98.348	98.493	74.841	74.83
RMSSD	ms	45.042	44.799	35.525	47.501	32.333	29.837	33.210	33.293	37.769	35.877	23.702	23.498
NN50	beats	119.000	110	30	49	21	8	20	20	61	50	1	0
pNN50	%	31.649	29.255	15.873	25.926	12.805	6.202	9.901	9.901	18.264	16.502	0.658	0
LF absolute power	ms^2^	531.203	547.240	390.920	542.448	1935.732	894.022	2088.129	1897.168	737.441	768.170	173.786	176.123
HF absolute power	ms^2^	551.371	556.553	236.603	532.964	118.777	73.803	611.976	577.538	762.765	641.054	236.525	228.844
LF relative power	%	46.648	47.134	60.275	48.829	92.804	90.974	72.203	71.653	48.502	53.302	42.004	43.126
HF relative power	%	48.419	47.936	36.482	47.975	5.695	7.510	21.161	21.813	50.167	44.481	57.168	56.036
LF normalized power	adim	48.857	49.357	62.186	50.249	94.218	92.362	77.316	76.658	49.149	54.487	42.350	43.486
HF normalized power	adim	50.712	50.197	37.638	49.371	5.781	7.625	22.659	23.336	50.837	45.471	57.639	56.504
Total power	ms^2^	1138.755	1161.030	648.557	1110.912	2085.821	982.719	2892.033	2647.701	1520.451	1441.175	413.735	408.389
LF/HF	adim	0.963	0.983	1.652	1.018	16.297	12.114	3.412	3.285	0.967	1.198	0.735	0.770
Poincaré SD1	ms	31.892	31.720	25.187	33.678	22.933	21.181	23.542	23.601	26.747	25.411	16.816	16.671
Poincaré SD2	ms	45.021	45.316	33.186	40.194	61.822	49.825	65.114	65.221	47.888	47.749	25.834	25.841
SD2/SD1	adim	1.412	1.429	1.318	1.194	2.696	2.352	2.766	2.764	1.790	1.879	1.536	1.550
Approximate entropy	a.u.	1.158	1.132	0.863	0.828	0.683	0.625	0.940	0.872	1.076	1.109	0.754	0.807
Sample entropy	a.u.	1.863	1.853	2.098	1.767	1.041	1.108	1.522	1.463	1.693	2.009	1.892	1.882
DFA α1	a.u.	0.925	0.931	0.985	0.869	1.358	1.121	1.430	1.450	1.021	1.024	0.863	0.874
DFA α2	a.u.	0.352	0.351	0.267	0.254	0.063	0.063	0.196	0.175	0.214	0.206	0.236	0.235

## References

[1] Fowler N.O. (1991). Precordial Palpation and Auscultation. Diagnosis of Heart Disease.

[2] Durand L.G., Pibarot P. (2017). Review: Most Recent Advancements in Digital Signal Processing of the Phonocardiogram. Crit. Rev. Biomed. Eng..

[3] Padilla-Ortiz A.L., Ibarra D. (2018). Lung and Heart Sounds Analysis: State-of-the-Art and Future Trends. Crit. Rev. Biomed. Eng..

[4] Hutchins J. (2015). Blood Pressure, Heart Tones, and Diagnoses. Handbook of Cardiac Anatomy, Physiology, and Devices.

[5] Littmann D. (1964). Heart sounds. Disease-a-Month.

[6] Greenstein J. (1955). Phonocardiography; its application to clinical medicine. S. Afr. Med. J..

[7] Muto V., Andreozzi E., Cappelli C., Centracchio J., Di Meo G., Esposito D., Bifulco P., De Caro D. (2023). Real-Time Implementation of a Frequency Shifter for Enhancement of Heart Sounds Perception on VLIW DSP Platform. Electronics.

[8] Jung D.K. (2021). Reinforcing Stethoscope Sound using Spectral Shift. J. Sens. Sci. Technol..

[9] Aumann H.M., Emanetoglu N.W. (2019). Stethoscope with digital frequency translation for improved audibility. Healthc. Technol. Lett..

[10] Ismail S., Siddiqi I., Akram U. (2018). Localization and classification of heart beats in phonocardiography signals—A comprehensive review. EURASIP J. Adv. Signal Process..

[11] Dimond E.G., Benchimol A. (1961). Phonocardiography. Calif. Med..

[12] Rappaport M.B., Sprague H.B. (1942). The graphic registration of the normal heart sounds. Am. Heart J..

[13] Boyer N.H., Eckstein R.W., Wiggers C.J. (1940). The characteristics of normal heart sounds recorded by direct methods. Am. Heart J..

[14] Sprague H.B., Ongley P.A. (1954). The clinical value of phonocardiography. Circulation.

[15] Vermarien H., Webster J.G. (2006). Phonocardiography. Encyclopedia of Medical Devices and Instrumentation.

[16] Giordano N., Knaflitz M. (2019). A Novel Method for Measuring the Timing of Heart Sound Components through Digital Phonocardiography. Sensors.

[17] Liu C., Springer D., Li Q., Moody B., Juan R.A., Chorro F.J., Castells F., Roig J.M., Silva I., Johnson A.E.W. (2016). An open access database for the evaluation of heart sound algorithms. Physiol. Meas..

[18] Bhoi A.K., Sherpa K.S., Khandelwal B. (2015). Multidimensional Analytical Study of Heart Sounds: A Review. Int. J. Bioautomation.

[19] Li S., Li F., Tang S., Xiong W. (2020). A Review of Computer-Aided Heart Sound Detection Techniques. BioMed Res. Int..

[20] Sathyanarayanan S., Murthy S., Chitnis S. (2023). A Comprehensive Survey of Analysis of Heart Sounds using Machine Learning Techniques to Detect Heart Diseases. J. Popul. Ther. Clin. Pharmacol..

[21] Chen W., Sun Q., Chen X., Xie G., Wu H., Xu C. (2021). Deep Learning Methods for Heart Sounds Classification: A Systematic Review. Entropy.

[22] Torre-Cruz J., Martinez-Muñoz D., Ruiz-Reyes N., Muñoz-Montoro A.J., Puentes-Chiachio M., Canadas-Quesada F.J. (2022). Unsupervised detection and classification of heartbeats using the dissimilarity matrix in PCG signals. Comput. Methods Programs Biomed..

[23] Kumar D., Carvalho P., Antunes M., Henriques J., Eugenio L., Schmidt R., Habetha J. Detection of S1 and S2 heart sounds by high frequency signatures. Proceedings of the 2006 International Conference of the IEEE Engineering in Medicine and Biology Society.

[24] Patidar S., Pachori R.B. (2013). Segmentation of cardiac sound signals by removing murmurs using constrained tunable-Q wavelet transform. Biomed. Signal Process Control.

[25] Moukadem A., Dieterlen A., Hueber N., Brandt C. (2013). A robust heart sounds segmentation module based on S-transform. Biomed. Signal Process Control.

[26] Ramović A., Bandić L., Kevrić J., Germović E., Subasi A., Badnjevic A. (2017). Wavelet and Teager Energy Operator (TEO) for Heart Sound Processing and Identification. CMBEBIH 2017: Proceedings of the International Conference on Medical and Biological Engineering 2017.

[27] Jaros R., Koutny J., Ladrova M., Martinek R. (2023). Novel phonocardiography system for heartbeat detection from various locations. Sci. Rep..

[28] Debbal S.M., Hamza L., Meziani F. (2021). Heartbeat cardiac sounds signals analysis by using the energy envelogram. J. Heart Vasc..

[29] Nath M., Srivastava S., Kulshrestha N., Singh D. (2021). Detection and localization of S1 and S2 heart sounds by 3rd order normalized average Shannon energy envelope algorithm. J. Eng. Med..

[30] Mondal A., Bhattacharya P., Saha G. (2013). An automated tool for localization of heart sound components S1, S2, S3 and S4 in pulmonary sounds using Hilbert transform and Heron’s formula. Springerplus.

[31] Bourouhou A., Jilbab A., Nacir C., Hammouch A. Detection and localization algorithm of the S1 and S2 heart sounds. Proceedings of the 2017 International Conference on Electrical and Information Technologies (ICEIT).

[32] Prasad R., Yilmaz G., Chetelat O., Doss M.M. Detection Of S1 And S2 Locations in Phonocardiogram Signals Using Zero Frequency Filter. Proceedings of the 2020 IEEE International Conference on Acoustics, Speech and Signal Processing (ICASSP).

[33] Liu Q., Wu X., Ma X. (2018). An automatic segmentation method for heart sounds. Biomed. Eng. Online.

[34] Belmecheri M.Z., Ahfir M., Kale I. (2018). Automatic heart sounds segmentation based on the correlation coefficients matrix for similar cardiac cycles identification. Biomed. Signal Process Control.

[35] Guven M., Uysal F. (2023). A New Method for Heart Disease Detection: Long Short-Term Feature Extraction from Heart Sound Data. Sensors.

[36] Boulares M., Alotaibi R., AlMansour A., Barnawi A. (2021). Cardiovascular Disease Recognition Based on Heartbeat Segmentation and Selection Process. Int. J. Environ. Res. Public. Health.

[37] Springer D.B., Tarassenko L., Clifford G.D. (2016). Logistic Regression-HSMM-Based Heart Sound Segmentation. IEEE. Trans. Biomed. Eng..

[38] Mubarak Q.U., Akram M.U., Shaukat A., Hussain F., Khawaja S.G., Butt W.H. (2018). Analysis of PCG signals using quality assessment and homomorphic filters for localization and classification of heart sounds. Comput. Methods Programs Biomed..

[39] Malik S.I., Akram M.U., Siddiqi I. (2019). Localization and classification of heartbeats using robust adaptive algorithm. Biomed. Signal Process Control.

[40] Afshan Z., Abid A., Hussain F. Localization of Phonocardiogram Signals Using Multi-level Threshold and Support Vector Machine. Proceedings of the 2019 IEEE International Symposium on Signal Processing and Information Technology (ISSPIT).

[41] Khan F.A., Abid A., Khan M.S. (2020). Automatic heart sound classification from segmented/unsegmented phonocardiogram signals using time and frequency features. Physiol. Meas..

[42] Xu X., Geng X., Gao Z., Yang H., Dai Z., Zhang H. (2023). Optimal Heart Sound Segmentation Algorithm Based on K-Mean Clustering and Wavelet Transform. Appl. Sci..

[43] Renna F., Martins M.L., Coimbra M. Joint Training of Hidden Markov Model and Neural Network for Heart Sound Segmentation. Proceedings of the 2021 Computing in Cardiology (CinC).

[44] Daponte P., De Vito L., Iadarola G., Picariello F., Rapuano S. Deterministic Compressed Sensing of heart sound signals. Proceedings of the 2021 IEEE International Symposium on Medical Measurements and Applications (MeMeA).

[45] Amiriparian S., Schmitt M., Cummins N., Qian K., Dong F., Schuller B. Deep Unsupervised Representation Learning for Abnormal Heart Sound Classification. Proceedings of the 2018 40th Annual International Conference of the IEEE Engineering in Medicine and Biology Society (EMBC).

[46] Andreozzi E., Fratini A., Esposito D., Naik G., Polley C., Gargiulo G.D., Bifulco P. (2020). Forcecardiography: A Novel Technique to Measure Heart Mechanical Vibrations onto the Chest Wall. Sensors.

[47] Andreozzi E., Gargiulo G.D., Esposito D., Bifulco P. (2021). A Novel Broadband Forcecardiography Sensor for Simultaneous Monitoring of Respiration, Infrasonic Cardiac Vibrations and Heart Sounds. Front. Physiol..

[48] Andreozzi E., Centracchio J., Punzo V., Esposito D., Polley C., Gargiulo G.D., Bifulco P. (2021). Respiration Monitoring via Forcecardiography Sensors. Sensors.

[49] Andreozzi E., Centracchio J., Esposito D., Bifulco P. (2022). A Comparison of Heart Pulsations Provided by Forcecardiography and Double Integration of Seismocardiogram. Bioengineering.

[50] Andreozzi E., Sabbadini R., Centracchio J., Bifulco P., Irace A., Breglio G., Riccio M. (2022). Multimodal Finger PulseWave Sensing: Comparison of Forcecardiography and Photoplethysmography Sensors. Sensors.

[51] Centracchio J., Andreozzi E., Esposito D., Gargiulo G.D., Bifulco P. (2022). Detection of Aortic Valve Opening and Estimation of Pre-Ejection Period in Forcecardiography Recordings. Bioengineering.

[52] Centracchio J., Esposito D., Gargiulo G.D., Andreozzi E. (2022). Changes in Forcecardiography Heartbeat Morphology Induced by Cardio-Respiratory Interactions. Sensors.

[53] Centracchio J., Andreozzi E., Esposito D., Gargiulo G.D. (2022). Respiratory-Induced Amplitude Modulation of Forcecardiography Signals. Bioengineering.

[54] Centracchio J., Parlato S., Esposito D., Bifulco P., Andreozzi E. (2023). ECG-Free Heartbeat Detection in Seismocardiography Signals via Template Matching. Sensors.

[55] Parlato S., Centracchio J., Esposito D., Bifulco P., Andreozzi E. (2023). Heartbeat Detection in Gyrocardiography Signals without Concurrent ECG Tracings. Sensors.

[56] Parlato S., Centracchio J., Esposito D., Bifulco P., Andreozzi E. (2023). ECG-Free Heartbeat Detection in Seismocardiography and Gyrocardiography Signals Provides Acceptable Heart Rate Variability Indices in Healthy and Pathological Subjects. Sensors.

[57] Sapira J.D. (1990). The Art and Science of Bedside Diagnosis.

[58] Savitzky A., Golay M.J.E. (1964). Smoothing and Differentiation of Data by Simplified Least Squares Procedures. Anal. Chem..

[59] Pan J., Tompkins W.J. (1985). A real-time QRS detection algorithm. IEEE Trans. Biomed. Eng..

[60] Sedghamiz H. (2018). BioSigKit: A Matlab Toolbox and Interface for Analysis of BioSignals. J. Open. Source Softw..

[61] Yoo J.C., Han T.H. (2009). Fast Normalized Cross-Correlation. Circuits Syst. Signal Process.

[62] Briechle K., Hanebeck U.D. Template matching using fast normalized cross correlation. Proceedings of the Society of Photo-Optical Instrumentation Engineers (SPIE) 4387, Optical Pattern Recognition XII.

[63] Chen Y.H., Chen H.H., Chen T.C., Chen L.G. Robust heart rate measurement with phonocardiogram by on-line template extraction and matching. Proceedings of the Annual International Conference of the IEEE Engineering in Medicine and Biology Society (EMBC).

[64] Passing H., Bablok W. (1983). A new biometrical procedure for testing the equality of measurements from two different analytical methods. Application of linear regression procedures for method comparison studies in clinical chemistry, Part I. J. Clin. Chem. Clin. Biochem..

[65] Altman D.G., Bland J.M. (1983). Measurement in medicine: The analysis of method comparison studies. Statistician.

[66] Giavarina D. (2015). Understanding Bland Altman analysis. Biochem. Med..

[67] Padoan A. (2023). Passing and Bablok Regression, MATLAB Central File Exchange. https://www.mathworks.com/matlabcentral/fileexchange/24894-passing-and-bablok-regression.

[68] Ran K. (2020). Bland-Altman and Correlation Plot, MATLAB Central File Exchange. https://www.mathworks.com/matlabcentral/fileexchange/45049-bland-altman-and-correlation-plot.

[69] Tarvainen M.P., Niskanen J.P., Lipponen J.A., Ranta-Aho P.O., Karjalainen P.A. (2014). Kubios HRV-heart rate variability analysis software. Comput. Methods Programs Biomed..

[70] Niskanen J.P., Tarvainen M.P., Ranta-Aho P.O., Karjalainen P.A. (2004). Software for advanced HRV analysis. Comput. Methods Programs Biomed..

[71] Tarvainen M.P., Ranta-Aho P.O., Karjalainen P.A. (2002). An advanced detrending method with application to HRV analysis. IEEE. Trans. Biomed. Eng..

[72] Lipponen J.A., Tarvainen M.P. (2019). A robust algorithm for heart rate variability time series artefact correction using novel beat classification. J. Med. Eng. Technol..

[73] Tarvainen M.P., Lipponen J., Niskanen J.-P., Ranta-Aho P.O. Kubios HRV (ver 3.1) USER’S GUIDE. http://www.kubios.com/downloads/Kubios_HRV_Users_Guide_3_1_0.pdf.

[74] Erne P. (2008). Beyond auscultation--acoustic cardiography in the diagnosis and assessment of cardiac disease. Swiss Med. Wkly..

[75] Schmidt S.E., Winther S., Larsen B.S., Groenhoej M.H., Nissen L., Westra J., Frost L., Holm N.R., Mickley H., Steffensen F.H. (2019). Coronary artery disease risk reclassification by a new acoustic-based score. Int. J. Cardiovasc. Imaging.

[76] Lehmacher J., Neumann J.T., Sörensen N.A., Goßling A., Schmidt S.E., Zeller T., Blankenberg S., Westermann D., Clemmensen P.M. (2023). Diagnostic performance of a device for acoustic heart sound analysis in patients with suspected myocardial infarction. Open Heart.

[77] Khoor S., Kovacs I., Fugedi K., Horvath G., Domijan E., Domijan M. Telemedicine digital phonocardiography: Cost-effective strategies in heart failure screening and monitoring. Proceedings of the 2007 Computers in Cardiology.

